# Biallelic Loss‐Of‐Function Variant in *ATP5ME* Is Associated With Severe and Early Onset Oxidative Phosphorylation Deficiency

**DOI:** 10.1002/jimd.70222

**Published:** 2026-07-05

**Authors:** Pranavi Hegde, Aakanksha Anand, Rita Rani, Namanpreet Kaur, Ami Shah, Shilpa Kulkarni, Janani Supraja Mallavaram, Raghavender Medishetti, Amoolya Kandettu, Huzail Shaikh, Shahyan Siddiqui, Purvi Majethia, Vivekananda Bhat, Periyasamy Radhakrishnan, Aarti Sevilimedu, Sanjiban Chakrabarty, Anju Shukla

**Affiliations:** ^1^ Department of Public Health Genomics Manipal School of Life Sciences, Manipal Academy of Higher Education Manipal India; ^2^ Department of Medical Genetics Kasturba Medical College, Manipal Academy of Higher Education Manipal India; ^3^ Center for Innovation in Molecular and Pharmaceutical Sciences, Dr. Reddy's Institute of Life Sciences, University of Hyderabad Campus, Gachibowli Hyderabad India; ^4^ Bai Jerbai Wadia Hospital for Children Mumbai India; ^5^ Department of Radiology NMC Royal Hospital, Dubai Investment Park Dubai UAE; ^6^ Center for Rare Disease Models, Dr. Reddy's Institute of Life Sciences, University of Hyderabad Campus Hyderabad India

**Keywords:** ATP synthase, *ATP5ME*, complex V deficiency, mitochondrial bioenergetics, mitochondrial encephalopathy, oxidative phosphorylation, OXPHOS, subunit e, zebrafish

## Abstract

ATP synthase (complex V) catalyzes ATP synthesis and is composed of the F_1_ catalytic sector and the F_0_ proton‐conducting sector. The e subunit of the F_0_ sector, encoded by *ATP5ME*, is essential for complex V dimerization and cristae organization; however, genetic variants in *ATP5ME* have not yet been implicated in human disease. We ascertained a 4‐year‐old male, born of a consanguineous marriage, who presented with neuroregression, feeding difficulty, spasticity, encephalopathy, bilateral sensorineural hearing loss and optic atrophy. Exome sequencing identified a biallelic 62 bp deletion, c.‐48_14del in *ATP5ME* (NM_007100.4; NC_000004.12: g.674234_674295del), spanning the upstream sequence, the 5′ untranslated region, and part of exon 1. Patient‐derived fibroblasts exhibited markedly decreased *ATP5ME* transcript and protein levels, accompanied by a reduction in the expression of complex I, IV, and V subunits. In vitro assays demonstrated reduced activities of complexes I, IV, and V, impaired mitochondrial respiration, reduced reactive oxygen species levels, decreased mitochondrial membrane potential, and reduced ATP levels. Additionally, *atp5me* knockout zebrafish demonstrated a severe developmental phenotype characterized by craniofacial defects, reduced locomotion, and decreased ATP levels. This was accompanied by reduced protein levels of complex II and IV subunits, mild decrease in mtDNA, and upregulation of genes associated with glycolysis and oxidative stress. All observed phenotypes were rescued by human *ATP5ME* mRNA complementation, thereby validating the pathogenicity of *ATP5ME* deficiency in vivo.

## 1 Introduction

Mitochondrial oxidative phosphorylation (OXPHOS) is the principal energy‐generating process in human cells, driven by a coordinated interplay of five multisubunit complexes (complexes I–V) embedded in the inner mitochondrial membrane (IMM), which are composed of 92 different structural proteins encoded by both nuclear and mitochondrial genomes [1].

Complex V, also known as ATP synthase, catalyzes the synthesis of ATP from ADP and inorganic phosphate, utilizing the proton gradient generated by the upstream respiratory chain complexes [2]. In humans, complex V is assembled from subunits encoded by both nuclear and mitochondrial DNA and consists of 29 subunits [3], of which defects in four subunits have been associated with a human disease viz. *ATP5F1A* [?combined oxidative phosphorylation deficiency 22, (MIM 616045, AR), mitochondrial complex V (ATP synthase) deficiency, nuclear type 4A (MIM 620358, AD), and ?mitochondrial complex V (ATP synthase) deficiency, nuclear type 4B, encephalopathic type (MIM 615228, AR)], *ATP5MC3* [dystonia, early‐onset, and/or spastic paraplegia (MIM 619681, AD)], *ATP5F1E* [mitochondrial complex V deficiency, nuclear type 3 (MIM 614053, AR)], *ATP5PO* [mitochondrial complex V deficiency, nuclear type 7 (MIM 620359, AR)].

Complex V is composed of two functional sectors, where the catalytic F_1_ sector synthesizes ATP in the mitochondrial matrix, and the membrane‐embedded F_0_ sector mediates proton translocation across the IMM to generate the proton motive force required for ATP production. *ATP5ME* encodes a membrane‐embedded F_0_ accessory subunit (subunit e) that contributes to the structural integrity of the F_0_ sector and the overall complex V [2, 3]. Although disruption of accessory F_0_ subunits is expected to impair complex V assembly and function, defects in any of these accessory subunits have not yet been associated with a human disorder. Here, we report an individual with a biallelic loss‐of‐function deletion in *ATP5ME*, presenting with an early‐onset neurodegenerative phenotype. We demonstrate the functional consequence of this variant on transcript and protein expression, mitochondrial bioenergetics, and OXPHOS function. Furthermore, we demonstrate the essential and conserved role of *ATP5ME* through a zebrafish knockout model in mitochondrial function and neurodevelopment.

## 2 Methodology

### 2.1 Participant Recruitment

We recruited the proband from an Indian origin third‐degree consanguineous family as part of an ongoing study on rare genetic disorders. Written informed consent approved by the institutional ethics committee was obtained from the family.

### 2.2 Genetic Testing

Genomic DNA was extracted from peripheral blood using the QIAamp DNA Blood Midi Kit (QIAGEN, USA). Singleton whole exome sequencing (WES) was performed using the TWIST Bioscience capture kit on an Illumina NextSeq platform (Illumina, USA). Variant prioritization was performed using a rare variant filtering strategy, as described previously [4] (Table S1). Validation and segregation analysis of the prioritized variant in the family were performed by Sanger sequencing.

### 2.3 Cell Culture

Dermal fibroblasts were obtained from biopsies of the proband (P1) and age and sex matched controls (C1 and C2). Fibroblast cells were cultured in Dulbecco's modified Eagle's medium (DMEM) (Gibco, USA) supplemented with 10% fetal bovine serum (FBS) (Gibco, USA). Fibroblasts were enzymatically passaged in 0.25% trypsin–EDTA (HiMedia, India). All the cultures were maintained at 37°C in a humidified incubator with 5% CO_2_. The culture medium was replenished every alternate day. The cells were used for up to four passages only.

### 2.4 RNA Analysis

Total RNA was isolated from skin fibroblasts using TRIzol reagent, followed by cDNA synthesis and quantitative real‐time polymerase chain reaction (qRT‐PCR) analysis of *ATP5ME* and *SOD2* expression.

### 2.5 Immunoblotting

Protein extraction from control and patient‐derived fibroblasts, and immunoblotting were performed as previously described [5, 6].

### 2.6 Mitochondrial Functional Assays

Activities of mitochondrial respiratory chain complexes I, IV, and V were measured as previously described [7, 8], and were normalized to citrate synthase activity. Intracellular ATP levels were quantified using a luciferase‐based assay (cat. no. A22066; Invitrogen, USA) according to the manufacturer's instructions [9]. Oxygen consumption rate (OCR) and extracellular acidification rate (ECAR) were measured using the Seahorse XF Cell Mito Stress Test (Agilent, USA). Mitochondrial membrane potential (MMP) and reactive oxygen species (ROS) were assessed using Tetramethylrhodamine methyl ester perchlorate (TMRM; cat. no. T668; Invitrogen, USA) and MitoSOX Red mitochondrial superoxide indicator (cat. no. M36005; Invitrogen, USA), respectively, and analyzed using fluorescence‐activated cell sorting (FACS) (Partec, Germany). In‐gel activity assays of respiratory chain complexes were performed using Blue Native PAGE (BN‐PAGE).

### 2.7 Animal Ethics Procedures and Approval

All experiments with zebrafish were done in a CCSEA (previously CPCSEA)‐approved zebrafish facility at Dr. Reddy's Institute of Life Sciences (1100/po/Re/s/07/CPCSEA) in Hyderabad, India. The facility also has US‐NIH OLAW assurance (F22‐00539). All procedures and protocols were reviewed and approved by the Institutional Animal Ethics Committee (Protocol approval DRILS/IAEC/AS/2021‐1). The “Guidelines for Experimentation on Fishes, 2021” published by CPCSEA was used as a reference.

### 2.8 Zebrafish Assays

F0 knockouts were generated as described previously [5]. Briefly, multiple guides targeting early exons (1–3) in the zebrafish *atp5me* genes were injected into single‐cell stage embryos to generate the F0 knockouts. A mix of four guides with no binding sites in the zebrafish genome was used as a control. A reduction in both *atp5me* transcripts was determined by qRT‐PCR, and protein level reduction was measured by immunoblotting. Development was monitored by bright field imaging and recorded at 5 dpf. Locomotion analysis was carried out at 5 dpf using the ZebraBox recording chamber and analyzed using the Zebralab software, with a standard 10‐min light–dark program (ViewPoint Life Sciences, Lyon, France). Rescue experiments were performed by co‐injecting 250 pg of in vitro transcribed human *ATP5ME* mRNA along with the Cas9‐RNP. Gene expression analysis, mtDNA copy number, protein measurements, and ATP level estimation were all conducted in 5 dpf larvae (10–30 pooled larvae/n, *n* = 3).

### 2.9 Statistical Analysis

Statistical analyzes were performed using ANOVA or unpaired Student's *t*‐test. Data are represented as mean ± SD and a *p*‐value less than 0.05 was considered statistically significant. Cell‐based assays were performed in three biological replicates, each with three technical replicates. The zebrafish experiments were performed in two to four sets of injectants, each with *n* > 70 per group.

Detailed methodologies for PCR, qRT‐PCR, immunoblotting, mitochondrial respiratory chain complex activity assays, intracellular ATP estimation, OCR, MMP and ROS analysis, mtDNA copy number analysis, BN‐PAGE, and zebrafish experiments are provided in the Supporting Information. Primers used for molecular and in vitro assays are listed in Table S3, and those used for in vivo assays are listed in Table S4.

## 3 Results

### 3.1 Clinical Findings

**FIGURE 1:**
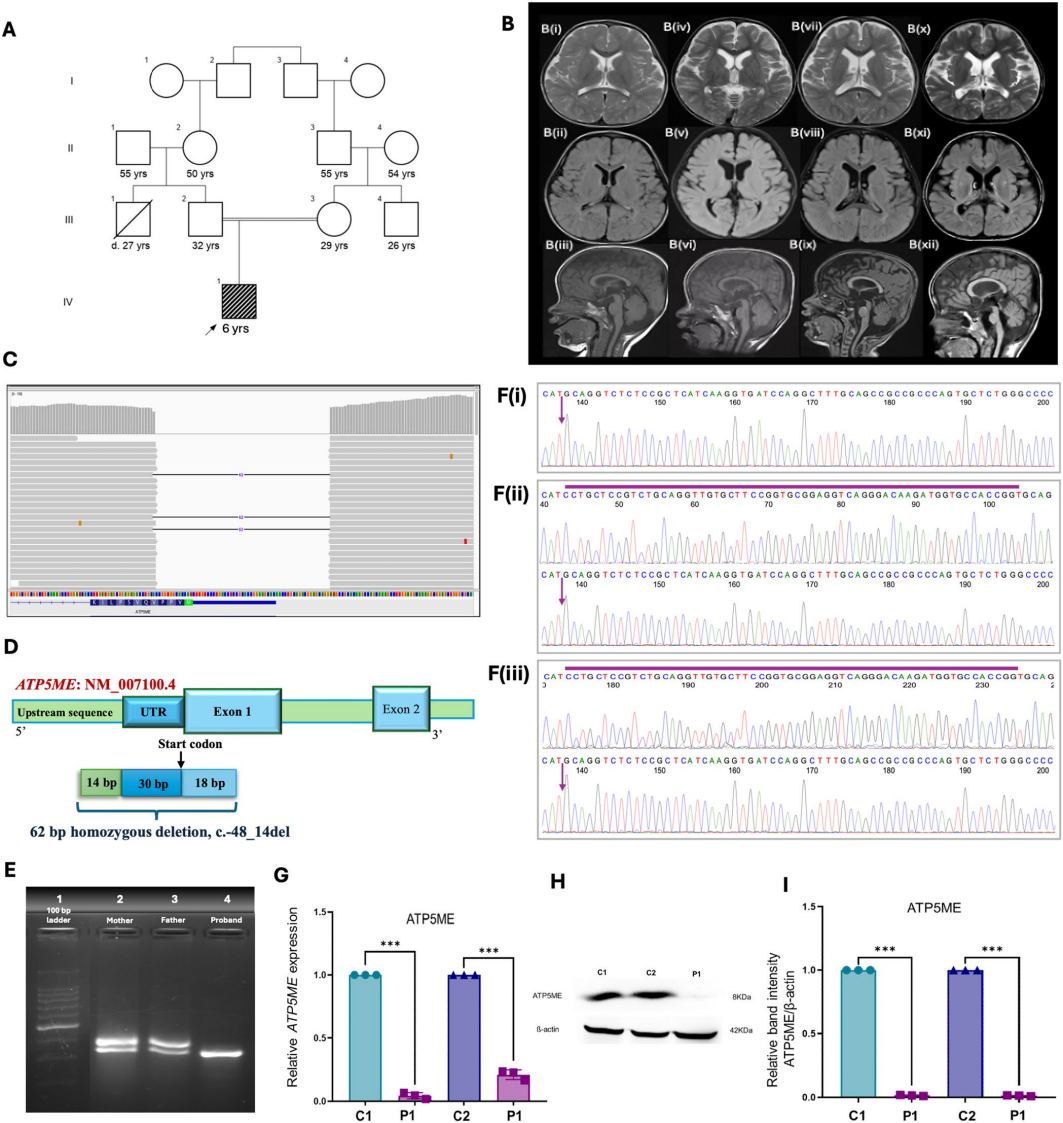
Genetic characterization and functional validation of the *ATP5ME* 62‐bp deletion (A) pedigree of the family. (B) Serial brain MRIs illustrating T2‐weighted axial, FLAIR axial, and T1‐weighted sagittal images at 2 years (Bi–iii), 3 years (Biv–vi), 3 years 6 months (Bvii–ix), and 4 years (Bx–xii) showing progressive neurodegeneration. (C) Integrated Genome Viewer visualization demonstrating the homozygous 62‐bp deletion (NC_000004.12: g.674234_674295del; NM_007100.4:c.‐48_14del) in *ATP5ME*. (D) Schematic representation of the 62‐bp deletion within *ATP5ME* (NM_007100.4). (E) PCR‐based detection of the *ATP5ME* 62‐bp deletion resolved on a 3% agarose gel. The unaffected mother (lane 2) and father (lane 3) show both wild‐type (~400‐bp) and deletion (~350‐bp) amplicons, consistent with heterozygosity. The proband (lane 4) shows a single ~350‐bp band, confirming a homozygous deletion. Lane 1 contains the 100‐bp molecular weight ladder. (F) Sanger sequencing chromatograms (i) 347‐bp allele harboring the 62‐bp deletion in proband; the site of deletion is indicated by a purple arrow. (ii) Maternal and (iii) paternal chromatograms showing the 409‐bp wild‐type allele and the 347‐bp deleted allele, confirming heterozygous carrier status. The 62‐bp sequence present in the wild‐type allele is highlighted by a horizontal purple bar. (G) qRT‐PCR analysis showing relative *ATP5ME* mRNA expression in C1, C2, and P1 cell lines. Expression of *ATP5ME* in P1 was significantly downregulated when compared to C1 and C2. *GAPDH* was used as the internal control. (H) Western blot analysis of ATP5ME protein expression in C1, C2, and P1 cell lines. (I) Densitometric analysis performed upon normalization of ATP5ME protein band intensity to the respective β‐Actin band. *Statistical significance: ***P < 0.001.

The proband is a first‐born male of third degree consanguineous parents with normal early development until 6 months of age, after which mild global developmental delay became evident (Figure 1A). At 13 months, he developed subacute, progressive neuroregression with gradual loss of previously acquired milestones, followed by progressive spasticity, dystonia, and seizures. Over time, he manifested progressive encephalopathy, feeding difficulties necessitating nasogastric support, and loss of visual and auditory responses. At the time of last evaluation at 4 years of age, he had growth failure, dysmorphic craniofacial features, axial hypotonia with generalized spastic‐dystonic quadriparesis, joint contractures, optic atrophy, and profound sensorineural hearing loss. Electroencephalography evolved to display multifocal epileptiform discharges with secondary generalization, consistent with epileptic encephalopathy (Figure S1A). While the magnetic resonance imaging (MRI) at 2 years of age was unremarkable (Figure 1Bi–iii), subtle posterior periventricular T2/FLAIR hyperintensities emerged by 3 years (Figure 1Biv–vi). On subsequent interval imaging, these abnormalities progressed, with increasing posterior white‐matter rarefaction and mild cerebral volume loss (Figure 1Bvii–ix). At 4 years, MRI showed posterior periventricular white‐matter hyperintensities, marked supratentorial atrophy, thalamic volume loss, mild pontine atrophy with preserved basal ganglia morphology (Figure 1Bx–xii). Magnetic resonance spectroscopy (MRS) demonstrated lactate peaks in the thalami (Figure S1B). Initial metabolic screening tests, including tandem mass spectrometry, urine organic acids, and blood lactate, were within normal limits. However, respiratory chain enzyme analysis done at an external lab on muscle tissue revealed markedly reduced activities (< 30%) of complexes I, III, and IV, consistent with a combined OXPHOS deficiency (Table S2 for detailed investigations).

### 3.2 Exome Sequencing Identifies a Biallelic Deletion in *ATP5ME*

Singleton exome sequencing revealed a biallelic 62 bp deletion in *ATP5ME* (NM_007100.4:c.‐48_14del; NC_000004.12:g.674234_674295del) in the proband. This deletion spans 18 bp of the upstream sequence, 30 bp of the 5′ untranslated region (UTR), and 14 bp of coding exon 1 (Figure 1C,D). Validation of this deletion using PCR revealed a single 347 bp product in the proband, consistent with a biallelic deletion. Both parents exhibited two bands (409 and 347 bp), corresponding to one wild‐type and one deleted allele (Figure 1E). Sanger sequencing confirmed the deletion in biallelic state in the proband and in heterozygous state in both parents (Figure 1F). This deletion is absent from the gnomAD database (v4.1.0) and has not been observed in our internal cohort of 4504 exomes. Following variant prioritization, no additional candidate variants consistent with the phenotype of the proband were identified. Mitochondrial genome sequencing in the proband and mother did not identify any known pathogenic variants.

### 3.3 In Vitro Analysis of the Deletion in *ATP5ME*

**FIGURE 2:**
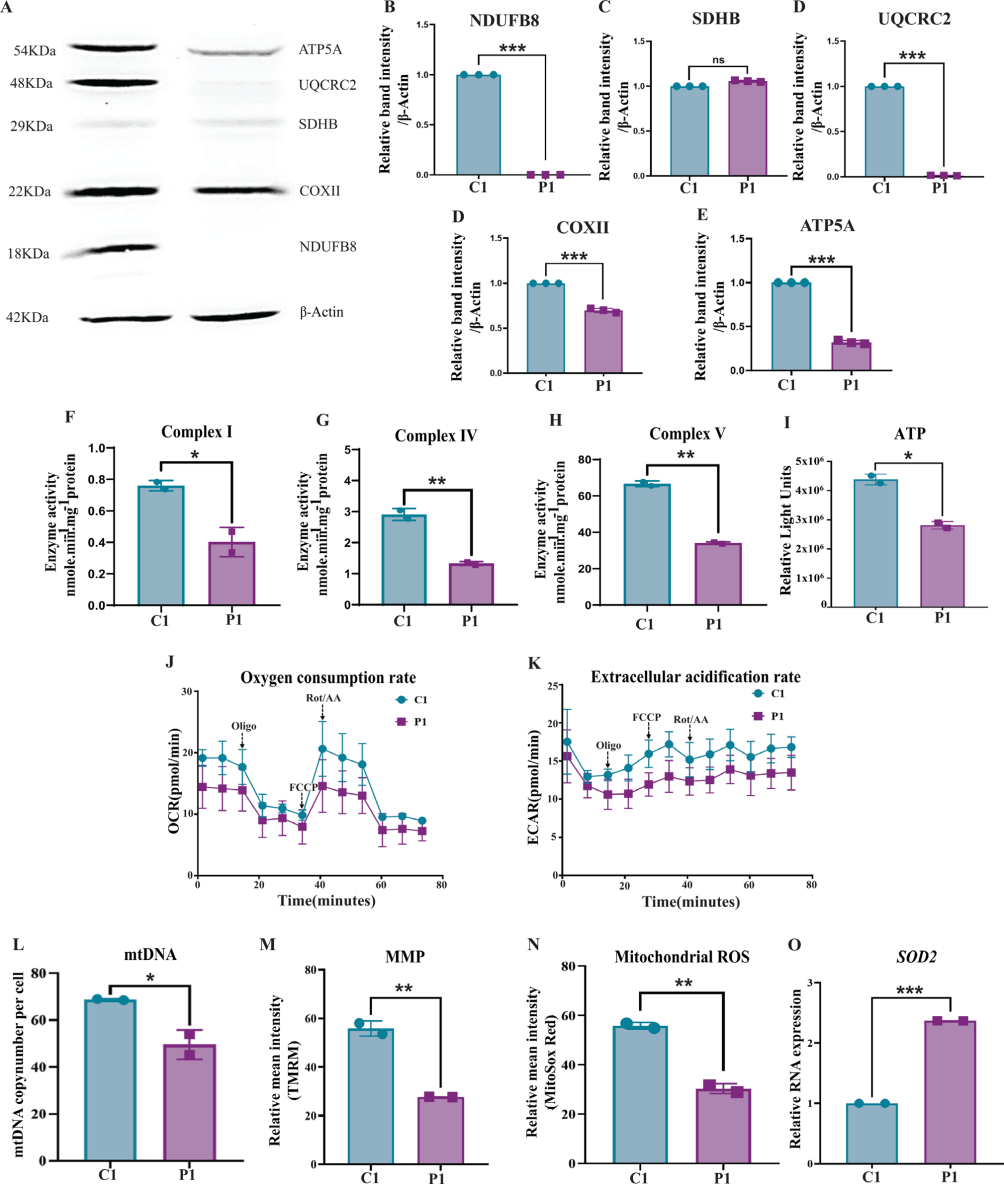
(A) Representative blot images for protein expression of OXPHOS proteins. Western blot analysis of protein expression of ATP5A, UQCRC2, SDHB, COX II, NDUFB8 in C1 and P1 cell lines; (B–F) Densitometric analysis was performed upon normalization of protein band intensity to respective β‐Actin band; (G) Mitochondrial complex I enzymatic activities in C1 and P1 cell lines, normalized to citrate synthase (CS) activity; (H) Mitochondrial complex IV enzymatic activities in control C1 and P1 cell lines, normalized to citrate synthase (CS) activity; (I) Mitochondrial complex V enzymatic activities in C1 and P1 cell lines, normalized to citrate synthase (CS) activity; (J) Representative graph showing intracellular ATP levels; (K) Representative graph showing the Oxygen Consumption Rate (OCR) in C1 and P1 cell lines. Injection of oligomycin, FCCP, antimycin A, and rotenone (A/R) is indicated; (L) Representative graph showing the Extracellular Acidification Rate (ECAR) in C1 and P1 cell lines. Injection of oligomycin, FCCP, and antimycin A and rotenone (A/R) are indicated; (M) Bar graph showing reduced mtDNA copy number in P1 cell lines; (N) Bar graph showing reduced relative fluorescence intensity of TMRM indicating decrease in mitochondrial membrane potential (MMP), respectively in P1 cell lines; (O) Bar graph showing the reduced relative fluorescence intensity of MitoSOX Red indicating decrease in mitochondrial reactive oxygen species (ROS), respectively in P1 cell lines due to defective OXPHOS activity; (P) Bar graph showing the increased relative expression of *SOD2* respectively in P1 cell lines, *p < 0.05, **p < 0.01, ***p < 0.001.

The functional consequence of the *ATP5ME*:c.‐48_14del deletion was investigated in primary skin fibroblasts derived from the proband (P1) alongside two matched controls (C1 and C2). qRT‐PCR analysis showed markedly reduced *ATP5ME* mRNA expression in P1 compared to controls (Figure 1G). Immunoblot analysis further confirmed decreased ATP5ME abundance in the patient‐derived fibroblasts (Figure 1H,I, Figure S2). Immunoblotting of representative mitochondrial OXPHOS subunits demonstrated reduced expression of proteins corresponding to complexes I, III, IV, and V (Figure 2A–F). Spectrophotometric enzyme assays performed on isolated mitochondrial fractions revealed decreased activities of complexes I, IV, and V in P1 (Figure 2G–I). In‐gel activity assays using BN‐PAGE further demonstrated reduced activity of mitochondrial respiratory chain complexes in P1 cells (Figure S3). Measurement of cellular ATP content demonstrated significantly reduced ATP levels in P1 compared to C1 (Figure 2J).

Mitochondrial respiration, assessed using the Seahorse XF24 Extracellular Flux Analyzer, showed an overall reduction in OCR along with ECAR in P1 cells, indicating impaired mitochondrial respiration (Figure 2K,L, Figure S4). mtDNA copy number analysis demonstrated reduced mtDNA content in P1 compared to C1 (Figure 2M). Flow cytometric analyzes revealed decreased MMP, measured using TMRM fluorescence, and reduced mitochondrial ROS levels, measured using MitoSOX Red, in P1 cells (Figure 2N,O). In addition, qRT‐PCR analysis showed increased *SOD2* mRNA expression in proband fibroblasts compared with controls (Figure 2P).

### 3.4 Impact of the Loss of the ATP5ME Orthologues in Zebrafish

**FIGURE 3:**
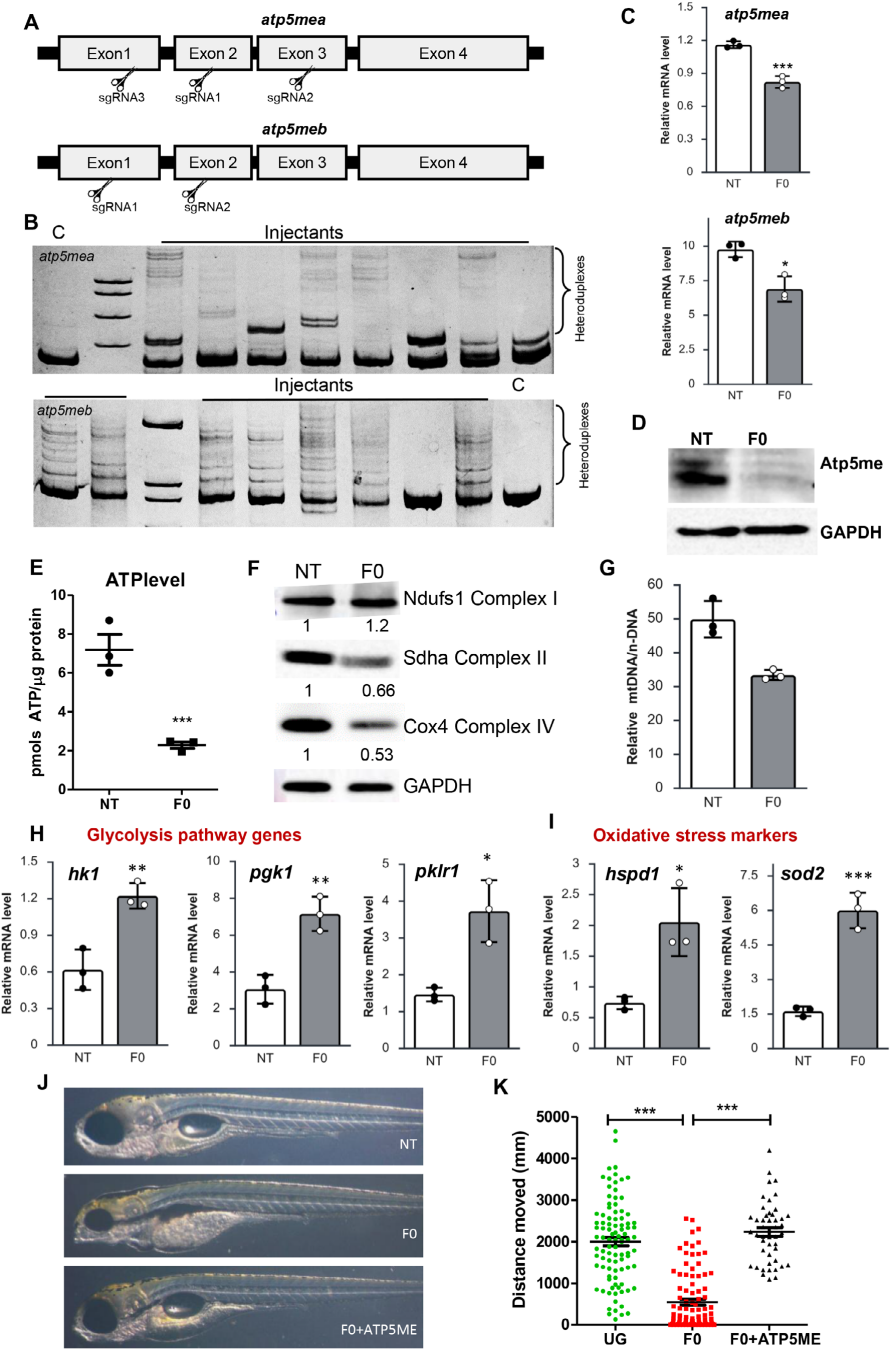
Generation and phenotypic characterization of the Atp5me crispant zebrafish. (A) A schematic of the zebrafish *atp5mea* and *atp5meb* genes with the location of the selected sgRNA target sites indicated. (B) Assessment of editing efficiency for sgRNA for each gene at 24 h postinjection, by a heteroduplex mobility assay (HMA). (C) Relative expression levels of the *atp5mea* and *b* mRNA in the controls (NT) and injectants (crispants, F0). (D) Atp5me protein level in crispants measured by immunoblotting. (E) Level of total ATP measured in larval lysate (5 dpf) in crispants relative to control using a standard kit. Results from 3 independent experiments are quantified, and error bars represent SEM. (F) Measurement of protein levels of the subunits of the OXPHOS complexes in crispants relative to control larvae by immunoblotting. Representative images are shown, relative quantification of band intensity from > 3 experiments is indicated under each blot. Charts are shown in Supporting Information (G) Quantification of mitochondrial DNA (mtDNA) in 5 dpf larvae (H) Relative mRNA levels of glycolysis pathways genes including *hk1, pgk1*, and *pklr1* and (I) oxidative stress markers *sod2* and *hspd1* in Atp5me crispants compared to controls. (J) Brightfield images of 5 dpf larvae from the indicated groups where crispants show yolk retention and delayed development, phenotypes rescued in the presence of *ATP5ME* supplementation. (K) Quantification of total distance moved in a standard locomotion analysis paradigm (10 min dark, 10 min light) comparing controls (*n* = 95), crispants (*n* = 95), and crispants with *ATP5ME* mRNA supplementation (*n* = 47) at 5 dpf.

There are two well‐conserved orthologues (> 60% identity at the protein level) of the *ATP5ME* gene in zebrafish, namely *atp5mea* and *atp5meb*. An F0 knockout strategy targeting both genes (Figure 3A) was used to mimic the near‐complete loss of protein observed in the patient, and the studies reported here were carried out in the F0 injectants (crispants). Upon injection of Cas9‐RNPs containing a mix of guides targeting both genes, significant levels of editing were observed in vivo, as measured by a heteroduplex mobility assay (HMA), as shown in representative images in Figure 3B. This was confirmed by a reduction in the levels of both transcripts (Figure 3C), as well as the Atp5me proteins, both of which are likely recognized by the antibody (Figures 3D and S5). A functional consequence of this loss of protein could be observed in terms of a drastic reduction in the amount of ATP in the larval lysate from the crispants as compared to the controls (Figure 3E).

Further, the crispants showed a reduction in the protein level of subunits from complexes II and IV (Figures 3F and S6), and a slight reduction in the mtDNA copy number (Figure 3G). As is expected and observed whenever there is a defect in mitochondrial ATP production, an increase in the expression of genes involved in the glycolysis pathway (*hk1, pgk1*, and *pklr1*) was detected in the crispants (Figure 3H). Additionally, a significant increase in the oxidative stress marker genes *hspd1* and *sod2* was also observed in the crispants, reflective of increased oxidative stress in the mitochondria (Figure 3I).

The Atp5me crispants showed a significant developmental delay, as seen from the images of 5 dpf larvae (Figure 3J), showing yolk retention, absent air sac inflation, small head and eye size, and jaw defects. This was accompanied by a severe locomotion defect, likely a result of the physical defects and the reduced ATP levels (Figure 3K). All the observed defects were rescued by complementation with mRNA encoding the human ATP5ME protein, confirming the specificity of the knockout phenotype (Figures 3J,K and S7A,B,C).

## 4 Discussion

We report an individual with a biallelic loss‐of‐function variant in *ATP5ME*, which encodes the e subunit of the F_0_ sector of mitochondrial complex V, and provide genetic and functional evidence that deficiency of this accessory subunit causes an early onset and severe mitochondrial disorder.

The proband presented with a progressive disorder with developmental delay, neuroregression, epileptic encephalopathy, oropharyngeal dysfunction, bilateral sensorineural hearing loss, optic atrophy, and spastic dystonia with hyperreflexia. These clinical findings overlap with the spectrum of other nuclear‐encoded complex V deficiencies, which often include early‐onset developmental delay, seizures, encephalopathy, spasticity, growth retardation, lactic acidosis, and poor survival. Serial neuroimaging demonstrated degenerative changes including cerebral, cerebellar, and thalamic atrophy accompanied by T2‐FLAIR hyperintensities in the deep white matter, a pattern overlapping with mitochondrial complex deficiency disorders [10, 11, 12, 13].

The biallelic 62 bp deletion, c.‐48_14del in *ATP5ME* is predicted to disrupt the regulatory region as well as the translation initiation site, a structural disruption expected to abolish normal transcription and translation. Consistent with this, patient‐derived fibroblasts exhibited markedly reduced *ATP5ME* transcript and protein levels. Loss of *ATP5ME*, an essential structural component required for complex V assembly, was reflected in impaired mitochondrial respiration, including reduced OCR, decreased ATP production, diminished MMP, and decreased ROS. These cellular findings aligned with the biochemical profile from muscle biopsy, which showed severe reductions in the enzymatic activities of complexes I, III, and IV. Decreased mtDNA copy number together with reduced activities of respiratory chain complexes I, IV, and V in patient‐derived fibroblasts suggested global perturbation of OXPHOS organization and function. Together, these results indicate that *ATP5ME* deficiency leads to destabilization of complex V with secondary respiratory chain defects and profound bioenergetic failure, providing a plausible basis for the proband's severe neurodegenerative phenotype. Although mitochondrial ROS levels were reduced in proband fibroblasts, brief or early mitochondrial ROS signals can activate antioxidant defense pathways, offering a likely explanation for increased SOD2 expression [14]. A similar rise in sod2 expression in atp5me crispants supports the activation of mitochondrial stress–responsive programs in the in vivo system as well.

*ATP5ME* encodes subunit e, a component required for complex V dimerization and higher‐order oligomerization within the IMM. In yeast, loss of subunit e is known to disrupt complex V dimer assembly [15, 16], while in bovine mitochondria, subunits e and g contribute to cristae curvature and the formation of ordered rows of complex V [17]. In human HAP1 cells, disruption of subunit e and other F_0_ accessory subunits has been shown to impair complex V dimerization and assembly, preserving the F_1_ catalytic core but leading to loss of membrane‐embedded F_0_ components [3, 18]. Impaired complex V dimerization and consequent defects in complex V assembly secondary to *ATP5ME* deficiency are also known to perturb cristae organization and lead to destabilization of respiratory supercomplexes. Loss of cristae integrity may then impair complexes I, III, and IV, while complex II is relatively preserved in such conditions, consistent with the biochemical profile observed in the proband [19, 20]. Reduced mtDNA copy number was also observed in both patient‐derived fibroblasts and atp5me crispants, which may additionally contribute to the combined OXPHOS deficiency observed in both in vitro and in vivo models. Ultrastructural analyzes were not performed in the present study, and therefore direct evidence of cristae disruption is lacking.

The subunit composition, structure, and function of complex V are highly conserved in zebrafish, mice, and humans. However, there are not many reports on complex V subunit knockouts or mutants in model organisms. In mice, the complex V α subunit knockout results in embryonic lethality (IKMC), as does the loss of TMEM70, a protein required for the biogenesis of the F_1_F_0_ complex V [21]. In zebrafish, morpholino mediated knockdown of *usmg5* (ATP5MK ortholog) resulted in reduced ventricular contraction and altered calcium signaling; however, no developmental phenotypes or mitochondrial features were reported [22]. Pharmacological inhibition of complex V using Oligomycin resulted in poor survival of larvae and delayed development [23], similar to the phenotypes observed in this study. Multiple mitochondrial features are apparent in the zebrafish Atp5me crispants. In addition to the expected drastic reduction in ATP levels and a decrease in mtDNA content, as previously reported in Atp5me knockout yeast [23], the loss of Atp5me appears to result in a reduction of at least two other complexes (II and IV), based on protein levels. The reduction in Sdha, a complex II subunit, is observed only in the zebrafish larval lysates, and is likely an indirect effect of the cristae remodeling and destabilization of inner membrane complexes, or a nonspecific consequence of increased turnover of dysfunctional mitochondria, or a species‐specific effect seen in this developmental window [24]. All of these findings point to a severe mitochondrial bioenergetic impairment, and although a compensatory increase in the expression of glycolysis pathway genes is observed, it is insufficient to restore ATP levels. Consequently, a severe global developmental delay is observed in crispant larvae, including a drastic locomotion defect, significantly small head size, and jaw defects, all of which directly correlate with the observed patient phenotypes of global as well as neurodevelopmental defects. The fact that these specific phenotypes are completely rescued by supplementation of the human *ATP5ME* mRNA indicates a direct causal contribution of Atp5me to these defects.

In conclusion, we report the first association of *ATP5ME* loss‐of‐function with a human mitochondrial disorder and provide genetic, functional, and in vivo evidence for its essential role in maintaining mitochondrial bioenergetics. Identification and delineation of unrelated individuals with variants in *ATP5ME* will further aid in validating this disease‐gene association.

## Author Contributions

Pranavi Hegde, Rita Rani, Aakanksha Anand, Amoolya Kandettu, Janani Supraja Mallavaram, and Raghavender Medishetti performed the experiments. Ami Shah, Shilpa Kulkarni, Aakanksha Anand, Vivekananda Bhat, and Anju Shukla were involved in the clinical evaluation of the proband. Namanpreet Kaur, Aakanksha Anand, Purvi Majethia, Periyasamy Radhakrishnan, and Anju Shukla analyzed and interpreted the clinical and genomic data. Rita Rani and Aarti Sevilimedu analyzed and interpreted the zebrafish data. Huzail Shaikh performed the bioinformatic analysis and Shahyan Siddique provided expert neuroradiology opinion. Aakanksha Anand and Pranavi Hegde drafted the manuscript. Sanjiban Chakrabarty, Aarti Sevilimedu, and Anju Shukla designed the experiments and supervised the study. All authors edited the paper with input from the other authors, approved the final manuscript as submitted, and agreed to be accountable for all aspects of the work.

## Funding

This work was supported by the DBT/Wellcome Trust India Alliance for the study, Centre for Rare Disease Diagnosis, Research and Training(IA/CRC/20/1/600002) and the Department of Health Research, India for the study, Delineating the genomic basis of neurodegeneration and mitochondrial disorders associated with defective DNA break repair (R.11014/33/2023‐GIA/HR).

## Ethics Statement

The study is approved by the institutional ethics committee, Kasturba Medical College, and Kasturba Hospital (IEC: 363/2020, IEC:78/2023). All experiments with zebrafish were done in a CCSEA (previously CPCSEA)‐approved zebrafish facility at Dr. Reddy's Institute of Life Sciences (1100/po/Re/s/07/CPCSEA) in Hyderabad, India. The facility also has US‐NIH OLAW assurance (F22‐00539). All procedures and protocols were reviewed and approved by the Institutional Animal Ethics Committee (Protocol approval DRILS/IAEC/AS/2021‐1). The “Guidelines for Experimentation on Fishes, 2021” published by CPCSEA was used as a reference.

## Consent

Informed consent was obtained from the recruited family prior to participation as part of an ongoing study on rare genetic disorders. The consent procedures were approved by the institutional ethics committee.

## Conflicts of Interest

The authors declare no conflicts of interest.

## Supporting information


**Table S1:** Summary of variant counts following bioinformatic filtering and prioritization in the study dataset.
**Table S2:** Summary of additional investigations in the proband.
**Table S3:** List of primer sequences used in in vitro assays.
**Table S4:** List of primer sequences used in in vivo assays.
**Figure S1:** (A) EEG showing multifocal epileptiform discharges with secondary generalization (B) Single‐voxel proton MRS of the thalamus at 4 years of age, demonstrating a lactate doublet at 1.3 ppm.
**Figure S2:** Representative uncropped immunoblots of (A) ATP5ME and (B) β‐Actin in C1, C2, and P1 cell lysates. The specific bands corresponding to ATP5ME and β‐Actin are highlighted.
**Figure S3:** Representative Blue Native PAGE (BN‐PAGE) in‐gel activity assays of mitochondrial respiratory chain complexes in C1 and P1 cell lines. (A) Coomassie blue staining showing markedly reduced abundance of Complex V holocomplex in P1 cells; (B) In‐gel Complex I activity assay performed by incubating the gel in 50 mM potassium phosphate buffer (pH 7.0) containing 0.2 mg/mL nitro blue tetrazolium (NBT) and 0.1 mg/mL NADH, demonstrating decreased Complex I activity in P1 cells; (C) In‐gel Complex IV activity assay performed using assay buffer containing diaminobenzidine (DAB) and reduced cytochrome c, demonstrating decreased Complex IV activity in P1 cells.
**Figure S4:** (A) Representative Seahorse instrument‐generated graph showing the OCR in control (C1) and patient (P1) cell lines. (B‐I) Representative bar graphs showing quantification of different mitochondrial bioenergetic parameters: basal respiration, proton leak, maximal respiratory capacity, ATP‐linked respiration, nonmitochondrial respiration, spare respiratory capacity (%), and coupling efficiency, *p < 0.05; *n* = 3.
**Figure S5:** Full images for Western blot experiments shown in Figure 3D (A) and F (B).
**Figure S6:** Quantification of the levels of OXPHOS complex subunits from at least three independent experiments (*N* = 3, *n* = 6 or 7) accompanying Figure 3F.
**Figure S7:** Rescue of phenotypes upon supplementation of human ATP5ME mRNA (A) Measurement of protein levels of the subunits of the OXPHOS complexes in control, crispants and crispants injected with *ATP5ME* mRNA, by immunoblotting. (B, C). Relative mRNA levels of glycolysis pathways genes and oxidative stress markers in control, Atp5me crispants and crispants injected with *ATP5ME* mRNA (*n* = 3/group).

## Data Availability

The data that support the findings of this study are available from the corresponding author upon reasonable request.
