# Supplementary material for: Biallelic Loss‐Of‐Function Variant in ATP5ME Is Associated With Severe and Early Onset Oxidative Phosphorylation Deficiency
Source: J Inherit Metab Dis. 2026 Jul 5;49(4):e70222. doi: 10.1002/jimd.70222 (PMC13334235; doi:10.1002/jimd.70222)
Supplement: Supplementary file 1 — Table S1: Summary of variant counts following bioinformatic filtering and prioritization in the study dataset. Table S2: Summary of additional investigations in the proband. Table S3: List of primer sequences used in in vitro assays. Table S4: List of primer sequences used in in vivo assays. Figure S1: (A) EEG showing multifocal epileptiform discharges with secondary generalization (B) Single‐voxel proton MRS of the thalamus at 4 years of age, demonstrating a lactate doublet at 1.3 ppm. Figure S2: Representative uncropped immunoblots of (A) ATP5ME and (B) β‐Actin in C1, C2, and P1 cell lysates. The specific bands corresponding to ATP5ME and β‐Actin are highlighted. Figure S3: Representative Blue Native PAGE (BN‐PAGE) in‐gel activity assays of mitochondrial respiratory chain complexes in C1 and P1 cell lines. (A) Coomassie blue staining showing markedly reduced abundance of Complex V holocomplex in P1 cells; (B) In‐gel Complex I activity assay performed by incubating the gel in 50 mM potassium phosphate buffer (pH 7.0) containing 0.2 mg/mL nitro blue tetrazolium (NBT) and 0.1 mg/mL NADH, demonstrating decreased Complex I activity in P1 cells; (C) In‐gel Complex IV activity assay performed using assay buffer containing diaminobenzidine (DAB) and reduced cytochrome c, demonstrating decreased Complex IV activity in P1 cells. Figure S4: (A) Representative Seahorse instrument‐generated graph showing the OCR in control (C1) and patient (P1) cell lines. (B‐I) Representative bar graphs showing quantification of different mitochondrial bioenergetic parameters: basal respiration, proton leak, maximal respiratory capacity, ATP‐linked respiration, nonmitochondrial respiration, spare respiratory capacity (%), and coupling efficiency, *p < 0.05; n = 3. Figure S5: Full images for Western blot experiments shown in Figure 3D (A) and F (B). Figure S6: Quantification of the levels of OXPHOS complex subunits from at least three independent experiments (N = 3, n = 6 or 7) accompanying Fi [file JIMD-49-0-s001.docx]

**Biallelic loss-of-function variant in *ATP5ME* is associated with severe and early onset oxidative phosphorylation deficiency**

Pranavi Hegde^1*^, Aakanksha Anand^2*^, Rita Rani^3^, Janani Supraja Mallavaram^3^, Namanpreet Kaur^2^, Ami Shah^4^, Shilpa Kulkarni^4^, Amoolya Kandettu^1^, Huzail Shaikh^2^, Shahyan Siddiqui^5^, Purvi Majethia^2^, Raghavender Medishetti^3^, Vivekananda Bhat^2^, Periyasamy Radhakrishnan^2^, Aarti Sevilimedu^3,6#^, Sanjiban Chakrabarty^1#^, Anju Shukla^2#^

*^1^Department of Public Health Genomics, Centre for DNA Repair and Genome Stability, Manipal*

*School of Life Sciences, Manipal Academy of Higher Education, Manipal, India*

*^2^Department of Medical Genetics, Kasturba Medical College, Manipal, Manipal Academy of Higher Education, Manipal, India*

*^3^Center for Innovation in Molecular and Pharmaceutical Sciences, Dr. Reddy’s Institute of Life*

*Sciences, University of Hyderabad Campus, Gachibowli, Hyderabad, India*

*^4^Bai Jerbai Wadia Hospital for Children, Mumbai, India*

*^5^Department of Radiology, NMC Royal Hospital, Dubai Investment Park, Dubai, United Arab Emirates*

*^6^Center for Rare Disease Models, Dr. Reddy’s Institute of Life Sciences, University of Hyderabad*

*Campus, Gachibowli, Hyderabad, India*

*Equally contributing first author

^#^Corresponding authors:

Dr Anju Shukla

Email id: [anju.shukla@manipal.edu](mailto:anju.shukla@manipal.edu)

ORCID: 0000-0001-8938-4941

Dr Sanjiban Chakrabarty

Email id: [sanjiban.c@manipal.edu](mailto:sanjiban.c@manipal.edu)

ORCID: 0000-0002-6018-8098

Dr Aarti Sevilimedu

Email id: [AartiS@drils.org](mailto:AartiS@drils.org)

ORCID: 0000-0003-2856-0213

**SUPPLEMENTARY METHODS**

**Sanger sequencing and reconditioning PCR**

Validation and segregation analysis of the biallelic 62 bp deletion was performed by Sanger sequencing in available family members. Primers spanning the deleted region were designed for PCR amplification (Supplementary Table S3). PCR amplification was carried out using GoTaq DNA Polymerase Master Mix (2X) (Promega, USA) in a total reaction volume of 25 µL containing ~100 ng genomic DNA, 1 µL each of forward and reverse primers (10 µM), and 1 µL of 4% DMSO. Cycling conditions comprised an initial denaturation at 95°C for 4 minutes, followed by 30 cycles of denaturation at 95°C for 30 seconds, annealing at 60°C for 30 seconds, and extension at 72°C for 40 seconds, with a final extension at 72°C for 7 minutes.

For reconditioning PCR, 1 µL of the purified primary PCR product diluted 1:10 was used as template for a fresh PCR reaction containing 12.5 µL GoTaq Master Mix, 0.5 µL each of forward and reverse primers (5 µM), and 1 µL of 4% DMSO in a final reaction volume of 25 µL. The reaction was subjected to 10 amplification cycles consisting of initial denaturation at 95°C for 3 minutes, followed by denaturation at 95°C for 40 seconds, annealing at 60°C for 30 seconds, and extension at 72°C for 50 seconds, without a final extension step. Reconditioning PCR was performed to minimise heteroduplex formation and reduce amplification artefacts prior to Sanger sequencing.

**cDNA conversion and qRT-PCR**

Total RNA was extracted from fibroblast cell lines derived from the proband (P1) and two controls (C1 and C2) using TRIzol Reagent (Invitrogen, cat. no. 15596026, USA) according to the manufacturer’s protocol. RNA concentration and purity were assessed spectrophotometrically by measuring absorbance at 260 nm and the A260/A280 ratio. For *ATP5ME* expression analysis, two µg of total RNA were reverse-transcribed into cDNA using the iScript gDNA Clear cDNA Synthesis Kit (Bio-Rad, cat. no. 1725035, USA) according to the manufacturer’s instructions. Quantitative real-time PCR (qRT-PCR) was performed on the QuantStudio 5 Real-Time PCR System (Applied Biosystems, USA) using 2× PowerUp SYBR Green Master Mix (Applied Biosystems, cat. no. A25776, USA) in a final reaction volume of 10 µL. Relative *ATP5ME* transcript levels were normalised to *GAPDH*. For *SOD2* expression analysis, two µg of total RNA were reverse-transcribed into cDNA using the High-Capacity cDNA Reverse Transcription Kit (Applied Biosystems, cat. no. 4374967, USA) according to the manufacturer’s instructions. qRT-PCR was performed under the same conditions as described above. Relative *SOD2* transcript levels were normalised to *ACTB*. All reactions were performed in technical triplicate across three independent biological replicates. Relative transcript quantification was carried out using the comparative cycle threshold (2^−ΔΔCt) method. [1] Primer sequences for *ATP5ME, SOD2, GAPDH*, and *ACTB* are provided in Supplementary Table S3.

**Immunoblotting**

Fibroblast lysates were prepared from control (C1 and C2) and proband (P1) fibroblast cell lines cultured to approximately 80% confluence. Cells were lysed in RIPA buffer (Sigma-Aldrich, USA) supplemented with a protease inhibitor cocktail to prevent proteolysis. Protein concentration was determined by the Bradford assay (Sigma-Aldrich, USA). Equal amounts of total protein (30–50 µg) were loaded on 15% SDS-polyacrylamide gels for resolving the low molecular weight ATP5ME protein (approximately 8 kDa). Following electrophoresis, proteins were transferred onto 0.45 µm nitrocellulose membranes (Bio-Rad, USA) using constant voltage. Membranes were blocked with 5% non-fat dry milk (HiMedia, India) prepared in a TBST buffer for 1 hour at room temperature. The membranes were then incubated overnight at 4°C with primary antibodies against *ATP5ME* (1:1000; Invitrogen, USA) and Total OXPHOS Human WB Antibody Cocktail (1:1000; Abcam, UK). After washing with TBST buffer, membranes were incubated with horseradish peroxidase (HRP)-conjugated secondary antibodies for 2 hours at room temperature. The secondary antibodies used were anti-rabbit (1:10000; Jackson ImmunoResearch Labs, USA) and anti-mouse (1:10000; Bio-Rad, USA). Protein bands were visualised using enhanced chemiluminescence substrate (Bio-Rad Laboratories, USA) and imaged on the iBright FL1500 imaging system (Invitrogen, USA). Densitometric quantification of protein bands was performed using ImageJ software (NIH, USA). ATP5ME and OXPHOS complex subunit levels were normalised to β-actin. Each experiment was performed in three independent biological replicates.

**Analysis of mitochondrial complex activity**

Mitochondrial respiratory chain enzyme activities for Complex I, IV, and V were measured as described previously. [2,3]

Complex I (CI; NADH:ubiquinone oxidoreductase) activity was measured spectrophotometrically by monitoring NADH oxidation at 340 nm using an Infinite 200 PRO multimode reader (Tecan, Switzerland). The assay was performed in a reaction mixture containing 100 mM potassium phosphate buffer (pH 7.5), sodium azide, antimycin A, fatty acid-free BSA, ubiquinone, and NADH. Isolated fibroblast mitochondria were subjected to three freeze–thaw cycles in hypotonic buffer prior to the assay to maximise enzymatic activity. The reaction was initiated by the addition of ubiquinone, and the decrease in absorbance at 340 nm was recorded. Specific Complex I activity was determined as the rotenone-sensitive activity by performing parallel reactions in the presence or absence of 1 mM rotenone. Complex I activity was normalised to citrate synthase activity.

Complex IV (CIV; cytochrome c oxidase) activity was determined spectrophotometrically by monitoring the oxidation of reduced cytochrome c at 550 nm using an Infinite 200 PRO multimode reader (Tecan, Switzerland). The assay mixture contained 100 mM potassium phosphate buffer (pH 7.0) and 1 mM reduced cytochrome c. The reaction was initiated by the addition of fibroblast lysate, and the decrease in absorbance at 550 nm was monitored for 3 minutes. Specificity of Complex IV activity was confirmed by inhibition with 10 mM sodium azide. Complex IV activity was normalised to citrate synthase activity.

Complex V (ATP synthase) activity was measured by monitoring ATP hydrolysis–coupled NADH oxidation at 340 nm using a Varioskan Multimode Microplate Reader (Thermo Fisher Scientific, USA). Specificity of Complex V activity was confirmed by inhibition with 10 µg/mL oligomycin. Complex V activity was normalised to citrate synthase activity.

**Oxygen consumption rate analysis**

Control (C1) and proband (P1) fibroblast cells were seeded at a density of 1 × 10⁴ cells per well in complete growth medium and incubated overnight at 37°C in 5% CO₂. Oxygen consumption rate (OCR) and extracellular acidification rate (ECAR) were measured using the Seahorse XF24 Extracellular Flux Analyzer (Agilent, USA). Prior to measurement, the culture medium was replaced with Seahorse XF assay medium supplemented with pyruvate, glutamine, and glucose, and the cells were incubated for 1 hour at 37°C without CO₂ equilibration. Sequential injections of oligomycin, FCCP, and rotenone/antimycin A were used to evaluate mitochondrial respiration and glycolytic flux.

**Intracellular ATP estimation**

Intracellular ATP levels were measured using the ATP Determination Kit (Invitrogen, cat. no. A22066, USA) according to the manufacturer’s instructions. Control (C1) and proband (P1) fibroblast cells (1 × 10⁶) were lysed in passive lysis buffer, and ATP levels were quantified from 5 µL of cell lysate using a luminometric assay. Luminescence was measured using a FB 12 Luminometer, and ATP levels were normalised to total protein concentration. Experiments were performed in triplicate across three independent biological replicates.

**Analysis of mitochondrial membrane potential and reactive oxygen species**

Mitochondrial membrane potential (MMP) and reactive oxygen species (ROS) levels were assessed using fluorescence-based flow cytometry. Control (C1) and proband (P1) fibroblast cells (4.5 × 10⁶) were seeded in 60 mm μ-Dishes (Ibidi, Germany) and cultured for 48 hours under standard conditions. For MMP measurement, cells were washed twice with PBS and stained with TMRM (100 nM; Invitrogen, USA) for 30 minutes at 37°C in the dark. For mitochondrial ROS estimation, cells were stained with MitoSOX™ Red (100 nM; Invitrogen, USA) under identical conditions. After staining, cells were immediately analysed by fluorescence-activated cell sorting (FACS) (Partec, Germany). Fluorescence intensity histograms were generated, and mean fluorescence intensities were quantified and compared between C1 and P1 cells using GraphPad Prism (version 8.0.1).

**mtDNA copy number analysis**

Genomic DNA was isolated from control (C1) and proband (P1) fibroblast cell lines using the PureLink Genomic DNA Mini Kit (Thermo Fisher Scientific, USA). Mitochondrial DNA (mtDNA) copy number analysis was performed by qRT-PCR using PowerUp SYBR Green Master Mix (Applied Biosystems, USA) on a QuantStudio real-time PCR system (Thermo Fisher Scientific, USA). The mitochondrial gene MT-CO2 was used as the mitochondrial target, and β-actin served as the nuclear reference gene. Relative mtDNA content was calculated using the comparative cycle threshold (2^−ΔΔCt) method. [4]

**Isolation and solubilization of mitochondrial proteins**

Mitochondrial protein fractions were isolated from fibroblast cell lines derived from the control (C1) and proband (P1) fibroblast cells. Approximately 400 µg of mitochondrial protein was resuspended in BN-PAGE solubilization buffer containing 50 mM imidazole-HCl (pH 7.0), 500 mM 6-aminohexanoic acid, and 1 mM EDTA. Membrane proteins were extracted using 20% digitonin at a detergent-to-protein ratio of 6 g/g protein and incubated on ice for 5 minutes. Samples were centrifuged at high speed for 10 minutes at 4°C, and the supernatant was collected. Subsequently, 5 µL of 50% glycerol and Coomassie Brilliant Blue G-250 were added at an approximate protein-to-dye ratio of 8:1 prior to electrophoresis.

**Blue Native PAGE**

Mitochondrial protein complexes were separated by Blue Native Polyacrylamide Gel Electrophoresis (BN-PAGE) using 4–16% gradient polyacrylamide gels prepared under cold conditions. Approximately 20 µL of solubilised mitochondrial protein sample was loaded per well. Electrophoresis was performed using native anode buffer in the lower chamber and dark cathode buffer containing Coomassie Brilliant Blue G-250 in the upper chamber. Gels were initially run at 100 V until samples entered the resolving gel, following which the cathode buffer was replaced with diluted cathode buffer (1:10 dilution), and electrophoresis was continued at 150 V until optimal separation of mitochondrial complexes was achieved. Following electrophoresis, the gel was stained with Coomassie Brilliant Blue G-250 to visualise the distribution of mitochondrial respiratory chain complexes. For in-gel Complex I activity analysis, BN-PAGE gels were incubated in a 50 mM potassium phosphate buffer (pH 7.0) containing 0.2 mg/mL nitro blue tetrazolium (NBT) and 0.1 mg/mL NADH. Active Complex I was visualised as a purple-blue precipitated band. Complex IV activity was assessed by incubating gels in 50 mM sodium phosphate buffer (pH 7.2) containing diaminobenzidine (DAB), cytochrome c, and sucrose. Catalytically active Complex IV was visualised as a brown precipitated band at the expected molecular weight position. [5]

***In vivo* studies**

**gRNA design, synthesis and microinjection**

Guide RNAs targeting exons 1-3 in *atp5mea* and *atp5meb*  genes, and genotyping primers, were designed using CHOPCHOP ([https://chopchop.cbu.uib.no](http://valenvm.cbu.uib.no/)) and Synthego ([https://design.synthego.com/#/)](https://design.synthego.com/#/). Single guide RNA (sgRNA) templates were ordered as DNA oligonucleotides with a T7 promoter at the 5′ end and a tail oligo at the 3′ end (Supplementary Table S4). DNA templates were amplified by PCR, and sgRNAs were synthesized using the Hi-Scribe™ T7 RNA synthesis kit (NEB). Editing efficiency of each guide was assessed 24 hours after injecting individual sgRNA-Cas9 complexes into one-cell stage embryos, by a standard heteroduplex mobility assay (HMA-PCR). Three guides targeting *atp5mea* and two targeting *atp5meb* (listed in Supplementary Table S4) were chosen to make the injection mix to generate the crispants in this study. For control microinjections, a 4-guide mix, consisting of four single guide RNAs with no binding sites in the zebrafish genome, was used.

To generate F0 knockout embryos, 5 nL of Cas9-sgRNA mix (containing 3 ng Cas9 protein and 2ng of multi-guide mix targeting *atp5me* genes (F0/crispants) or non-targeting guides (NT)) was injected in one-cell stage zebrafish embryos. Injected embryos were maintained at 28ºC in E3 medium, and monitored daily for developmental progress up to 5 days post-fertilization (dpf). Phenotypes were observed and imaged at 5 dpf using bright-field microscopy (EVOS cell imaging system) using 4x magnification and as per published methodology.[6]

**Rescue with human *ATP5ME* mRNA**

The full-length coding sequence of the human *ATP5ME* was amplified from cDNA isolated from HEK293T cells, using gene-specific primers, and cloned into a standard T7-promoter driven *in vitro* transcription (IVT) vector. Capped, polyadenylated mRNA was synthesized from this vector using the mMESSAGE mMACHINE T7 Transcription Kit (Invitrogen, AM1344). Following DNase treatment, RNA was purified using the RNA Clean & Concentrator-5 Kit (Zymo Research,

USA) and quantified. For rescue experiments, 250 pg of mRNA was co-injected with the Cas9-RNP into one-cell stage embryos.

**Quantitative real-time PCR analysis**

Total RNA was extracted from the 5 dpf larvae (30 pooled larvae/n) using Trizol reagent. cDNA was synthesized with 1 µg of RNA using the PrimeScript™ RT reagent Kit (RR037A-Takara). Real time PCR was performed using TB Green Premix Ex Taq II (Tli RNase H Plus) (TakaRa) in a QuantStudio5 machine. Relative gene expression levels were calculated using the comparative Ct method (ΔΔCt). RNAPD (*polr2d*) was used as the internal reference gene for normalization.

**Immunoblotting**

Ten zebrafish larvae were collected at 5 days post-fertilization (dpf) and homogenized in RIPA lysis buffer supplemented with protease inhibitors. Protein concentration was determined by the Bicinchoninic acid (BCA) method. Equal amounts of protein (
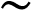
30 μg) were resolved on the 10% SDS-PAGE gel and transferred to the methanol-activated PVDF membrane (Millipore). Membranes were blocked and incubated with primary antibodies (dilution range: 1:1000–1:5000), followed by secondary antibodies (1:3000–1:5000). Signal detection was performed using the ECL substrate (Takara Bio), and images were captured using the Azure Biosystems imaging platform. Antibodies: ATP5ME (Proteintech.16483-1AP), COX4 (CST, 4850S), GAPDH (CST, 2118S), NDUFS1 (Proteintech, 12444-1AP), SDHA (Abclonal, AB13852). Images of full blots are shown in Supplementary Figure S5.

**ATP assay protocol**

For ATP extraction, 50 µg of larval lysate protein from each sample was subjected to phenol-TE/chloroform/deionized water treatment to release protein-bound ATP into the aqueous phase. ATP levels in the aqueous fraction were then measured using the Promega ENLITEN ATP Assay Kit, following the manufacturer’s instructions.

**mtDNA quantification**

Genomic DNA was extracted from 10-20 larvae using a Genomic DNA extraction kit (Purelink, Invitrogen) and diluted to 5 ng/µl. Using 1 µl genomic DNA as input, qPCR was set up using primers specific to mt-DNA and nuclear DNA (mt-nd1 and polg), with 3 technical replicates. The ratio of mtDNA/n-DNA was calculated using the ΔCt method.

**Locomotion**

Studies were performed on 5 dpf larvae by monitoring the larval locomotor activity during a 20-minute period using ZebraBox recording chamber (ViewPoint Life Sciences, Lyon, France). Larvae were gently pipetted into a 24-well plate 1 hour before the start of the experiment. The 24 well plate was placed inside the Zebrabox, and larvae were habituated for 10 minutes before the recording began. The ZebraLab software (Viewpoint Life Sciences, Lyon, France) was used to set up a program consisting of a 10-minute light period followed by a 10-minute dark period, with a one-minute integration period. Distance moved in the light period, dark period, and total distance moved in both periods were calculated and reported as Mean ± SEM. Statistical analysis was done using GraphPad Prism (one-way analysis of variance (ANOVA)).

**SUPPLEMENTARY TABLES**

**Supplementary Table S1: Summary of variant counts following bioinformatic filtering and prioritisation in the study dataset**

| **Initial filtering step** | **Number of variants** |
| --- | --- |
| Rare variants (population frequency <2%) | 4230 |
| Exonic and splice-site variants | 2441 |
| Rare variants absent in gnomAD v4.1 in heterozygous and homozygous state | 142 |
| Predicted deleterious variants | 13 |
| Variants consistent with the observed phenotype | 1 (*ATP5ME*:c.-48_14del) |

**Supplementary Table S2: Summary of additional investigations in the proband**

| **Date** | **Investigation** | **Result** | **Reference** |
| --- | --- | --- | --- |
|  | | | |
| November 2020 | Auditory evoked potentials | Bilateral low amplitude waves IV and V despite high stimulus intensity | |
|  | | | |
| January 2021 | Haemoglobin | 11.2 g/dL | 14-17 g/dL |
|  | Total leukocyte count | 11,800/µL | 5000-13000/ µL |
|  | Lymphocytes | 68% | 45%-65% |
|  | Neutrophils | 30% | 20%-60% |
|  | Platelet count | 4.15 lakhs/µL | 1.5-4.5 lakhs/µL |
|  | Thyroid-stimulating hormone | 6.08 mIU/L | 0.36–7.6 mIU/L |
|  | Mitochondrial genome sequencing (proband) | Homoplasmic variant of uncertain significance in *MT-ATP6* | |
|  | | | |
| April 2021 | Nerve conduction studies | Normal | |
|  | Cerebrospinal fluid (CSF) lactate | 2.87 mmol/L | 1.1–2.8 mmol/L |
|  | CSF protein | 22 mg/dL | 15-45 mg% |
|  | CSF glucose | 64 mg/dL | 45-80 mg% |
|  | CSF cells | Acellular | 0-16 / µL |
|  | CSF homocysteine | 12.18 µmol/L | <10 µmol/L |
|  | Blood glucose | 87 mg/dL | 65-180 mg/dL |
|  | Bone marrow aspirate | Normocellular | |
|  | Ultrasound abdomen | Normal | |
|  | Computed tomography brain | Mild prominence of ventricles and cortical sulci with increased extra-cerebral cerebrospinal fluid spaces | |
|  | | | |
| October 2021 | Muscle biopsy (histology and enzyme histochemistry) | Normal | |
|  | Total bilirubin | 0.22 mg/dL | <1.2 mg/dL |
|  | Aspartate aminotransferase | 33 U/L | <40 U/L |
|  | Alanine aminotransferase | 21 U/L | <41 U/L |
|  | Alkaline phosphatase | 213 U/L | 145-335 U/L |
|  | Total protein | 7.6 g/dL | 6-8 g/dL |
|  | Albumin | 5.2 g/dL | 3.8-5.4 g/dL |
|  | Urea | 26 mg/dL | 16-48 mg/dL |
|  | Creatinine | 0.3 mg/dL | 0.2-0.4 mg/dL |
|  | Serum sodium | 138 mmol/L | 136-145 mmol/L |
|  | Serum potassium | 4.88 mmol/L | 3.5-5.1 mmol/L |
|  | Serum chloride | 101.2 mmol/L | 98-107 mmol/L |
|  | CSF and serum autoimmune encephalitis panel | Anti-NMDA, AMPA1, AMPA2, CASPR, LGI1, GABAB1/B2: Negative | |
|  | | | |
| November 2021 | Two-dimensional echocardiography | Normal | |
|  | Serum paraneoplastic neuronal antibody panel | Anti-Hu, Ri, Yo, CV2: Negative | |
|  | | | |
| May 2022 | Karyotype | 46, XY | |
| June 2022 | Mitochondrial genome sequencing (mother) | Homoplasmic variant of uncertain significance in *MT-ATP6* | |
| March 2023 | Reanalysis of WES data (external laboratory) | No additional causative variants identified | |

**Supplementary Table S3: List of primer sequences used in *in vitro* assays**

| **No** | **Name** | **Sequence** |
| --- | --- | --- |
| 1. | ATP5ME_FP | ACTCCCCAGCCTTTGTGC |
| 2. | ATP5ME_RP | GTAGGCCACACCGAGGAAC |
| 3. | qATP5ME_FP | GGTCTCTCCGCTCATCAAGC |
| 4. | qATP5ME_RP | GCCAATTCTCTGGCAATCCG |
| 5. | qSOD2_FP | AAGACTCGAGATTGTTACTACAGATAATGC |
| 6. | qSOD2_RP | TACAGTCGACCAGTAATCTTACATGACACAT |
| 7. | qGAPDH_FP | ACCTGCCAAATATGATGAC |
| 8. | qGAPDH_RP | TCATACCAGGAAATGAGCTT |
| 9. | qACTB_FP | ATGATCTGGGTCATCTTCTC |
| 10. | qACTB_RP | CGGGCAGTCAAGGTTTTACA |
| 11. | qmMT-CO2_FP | CCTGCGACTCCTTGACGTTG |
| 12. | qmMT-CO2_RP | AGCGGTGAAAGTGGTTTGGTT |
| 13. | qmACTB_FP | TCACCCACACTGTGCCCATCTACG |
| 14. | qmACTB_RP | CAGCGGAACCGCTCATTGCCAATG |

**Supplementary Table S4: List of primer sequences used in *in vivo* assays**

| **No.** | **Name** | **Sequence** |
| --- | --- | --- |
| 1. | atp5mea sg1 | TAATACGACTCACTATAGGAGATGGTCTGCCTTGCTCATG TTTTAGAGCTAGAA |
| 2. | atp5mea sg2 | TAATACGACTCACTATAGGCTCCTCTCTTCAGCAGCAATG TTTTAGAGCTAGAA |
| 3. | atp5mea g3 | TAATACGACTCACTATAGGATAAGAGGGGAGACCTGTAC GTTTTAGAGCTAGAA |
| 4. | atp5meb Ex 1 sg1 | TAATACGACTCACTATAGGAGTGGCGACACTTGCACTGGG TTTTAGAGCTAGAA |
| 5. | atp5meb Ex 2 sg2 | TAATACGACTCACTATAGG  AACGAGCAACGCAGACCACCGTTTTAGAGCTAGAA |
| 6. | atp5mea FP | CGCTGAGTGCTTCATGATGTC |
| 7. | atp5mea RP | GCGCTCTTGTTCTTCCCTCA |
| 8. | atp5meb FP1 | ACGTCAGCGCGCTCATATAA |
| 9. | atp5meb RP1 | CCAACAGACAGCAACGCTTC |
| 10. | atp5meb FP2 | GTGCAGCGAAATGGTTCCTC |
| 11. | atp5meb RP2 | TCATCTGAGCGCCTGTCAAA |
| 12. | atp5mea qPCR FP | AGGGACAACAGACTGACAGC |
| 13. | atp5mea qPCR RP | CTCTCTTCAGCAGCAATGGG |
| 14. | atp5meb qPCR FP | CGCCACTGATTAAGACTGCC |
| 15. | atp5meb qPCR RP | TTTGGGCAGCTCATTTCAGG |
| 16. | ATP5ME IVT FP | CAACCTCAAACAGACACCGGGCCACCATGGTGCCACCGGTGCAGGTC |
| 17. | ATP5ME IVT RP | TTAATAGAAATTGGACAGCAAGAAAGGTCACTTTAATATGCTGTCATCTTCTG |
| 18. | hk1 FP | AGAAGACATCATCCACGGC |
| 19. | hk1 RP | CGTTTCGTCCATGTCAGCAA |
| 20. | pgk1 FP | AGAACATGGAGATCGGCAC |
| 21. | pgk1 RP | GCCTGTTGTTGCCTTCTCAT |
| 22. | pklr_FP | GGTGGAGACTCTGACCTCTG |
| 23. | pklr_RP | CTTCTTCAGCACGCGTGTTA |
| 24. | hspd1 FP | GGAGTCATGATGGCCGTAG |
| 25. | hspd1 RP | CACCCTTACGACCGACTT |
| 26. | sod2 FP | CGTGACTTTGGCTCATTTCA |
| 27. | sod2 RP | GCTGACATTCTCCCAGTTTAC |
| 28. | rnapd FP | CCAGATTCAGCCGCTTCAAG |
| 29. | rnapd RP | CAAACTGGGAATGAGGGCTT |
| 30. | POLG FP | GCGATTGAGAGCGTCTATAA |
| 31. | POLG RP | GTGACATGCAAAGCTCTAAC |
| 32. | ND1 FP | AGCCTACGCCGTACCAGTATT |
| 33 | ND1 RP | GTTTCACGCCATCAGCTACTG |

**SUPPLEMENTARY FIGURES WITH LEGENDS**


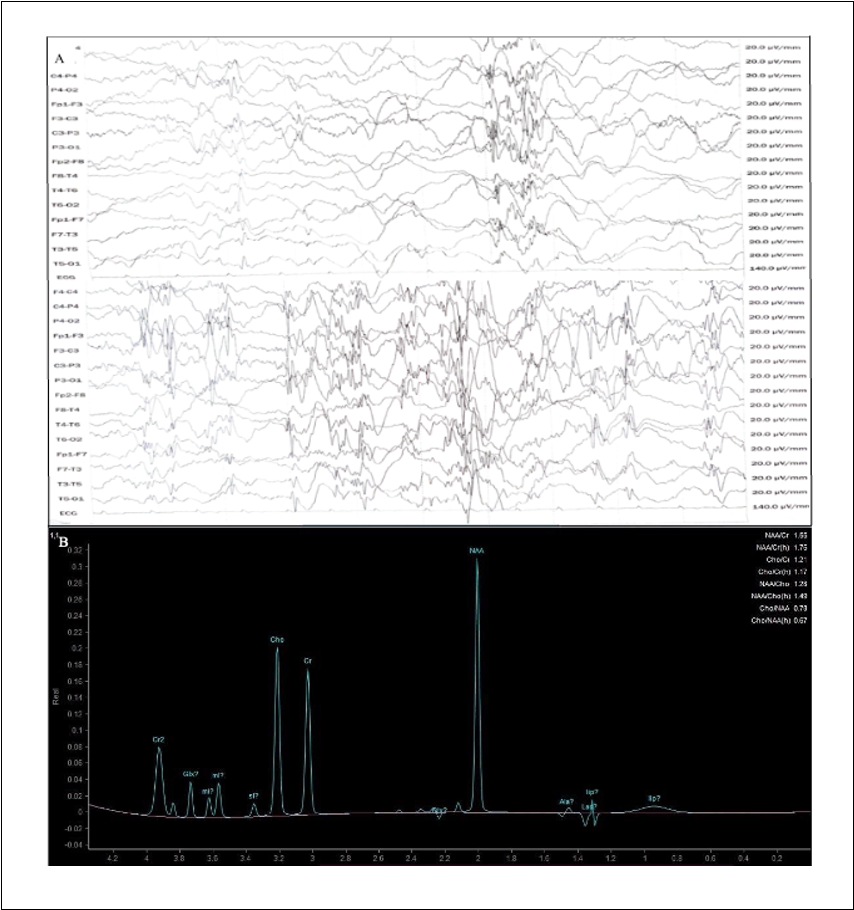


**Supplementary Figure S1: (A)** EEG showing multifocal epileptiform discharges with secondary generalisation **(B)** Single-voxel proton MRS of the thalamus at four years of age, demonstrating a lactate doublet at 1.3 ppm


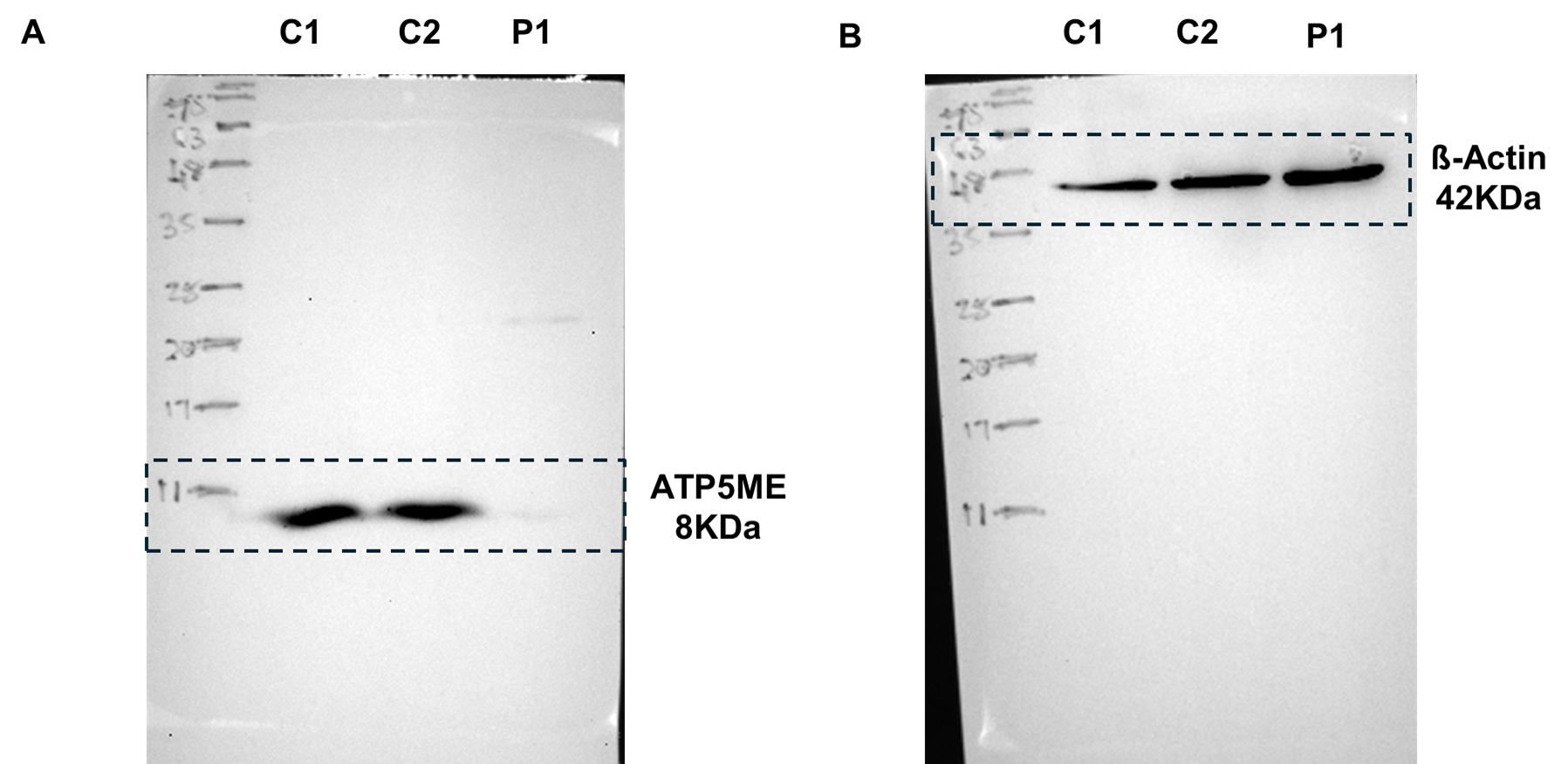


**Supplementary Figure S2:** Representative uncropped immunoblots of **(A)** ATP5ME and **(B)** β-Actin in C1, C2, and P1 cell lysates. The specific bands corresponding to ATP5ME and β-Actin are highlighted.


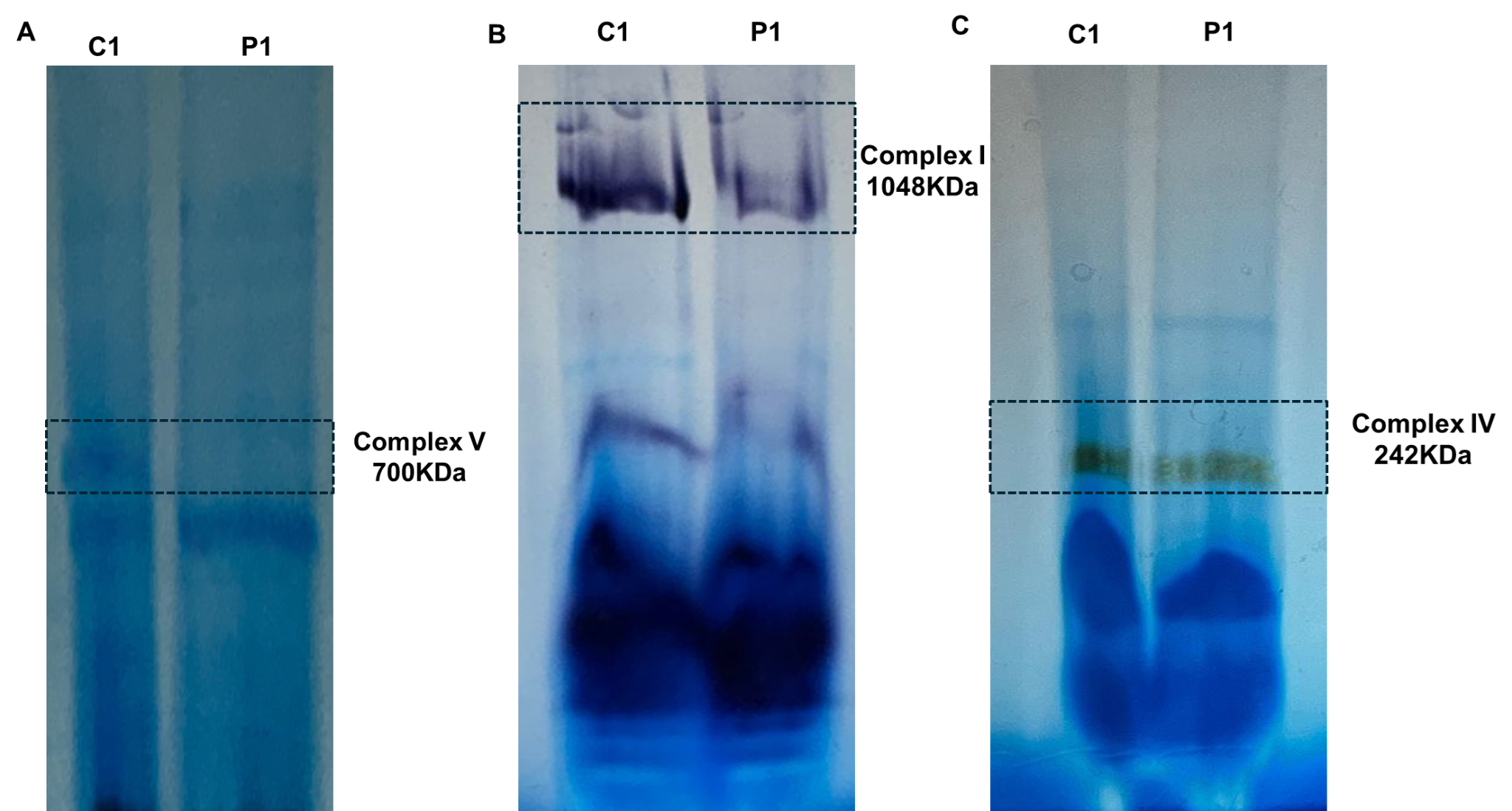


**Supplementary Figure S3:** Representative Blue Native PAGE (BN-PAGE) in-gel activity assays of mitochondrial respiratory chain complexes in C1 and P1 cell lines. **(A)** Coomassie blue staining showing markedly reduced abundance of Complex V holocomplex in P1 cells; **(B)** In-gel Complex I activity assay performed by incubating the gel in 50 mM potassium phosphate buffer (pH 7.0) containing 0.2 mg/mL nitro blue tetrazolium (NBT) and 0.1 mg/mL NADH, demonstrating decreased Complex I activity in P1 cells; **(C)** In-gel Complex IV activity assay performed using assay buffer containing diaminobenzidine (DAB) and reduced cytochrome c, demonstrating decreased Complex IV activity in P1 cells.


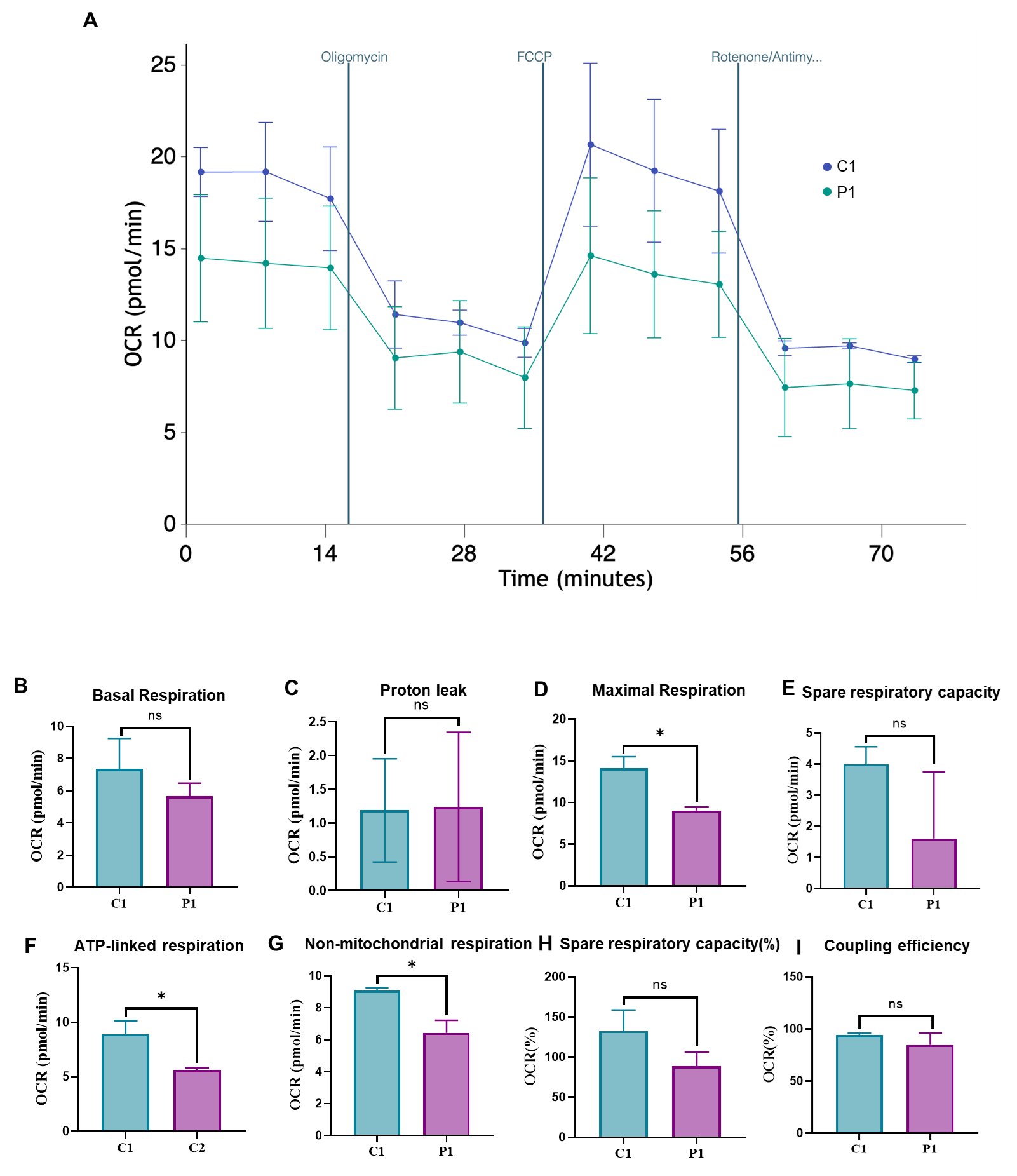


**Supplementary Figure S4: (A)** Representative Seahorse instrument-generated graph showing the OCR in control (C1) and patient (P1) cell lines. **(B-I)** Representative bar graphs showing quantification of different mitochondrial bioenergetic parameters: basal respiration, proton leak, maximal respiratory capacity, ATP-linked respiration, non-mitochondrial respiration, spare respiratory capacity (%), and coupling efficiency,*p < 0.05; n=3.


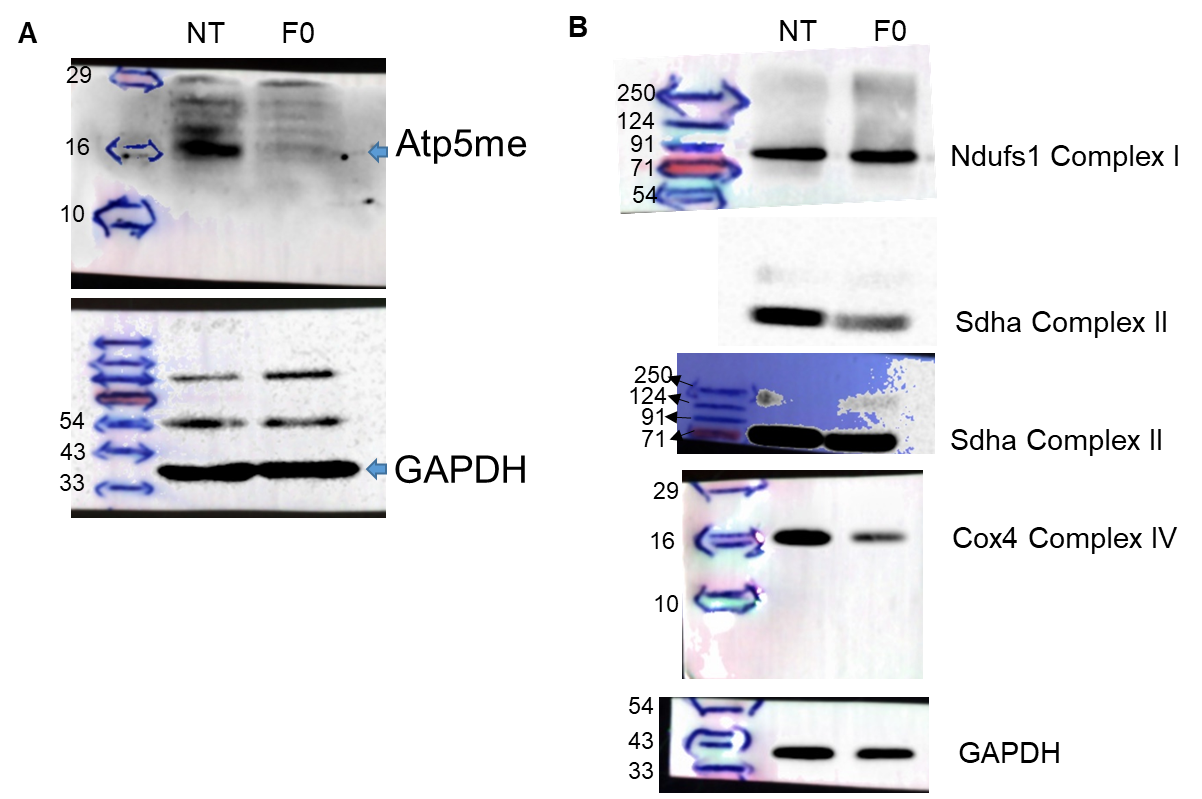


**Supplementary Figure S5:** Full images for Western blot experiments shown in Figure 3D **(A)** and F **(B)**


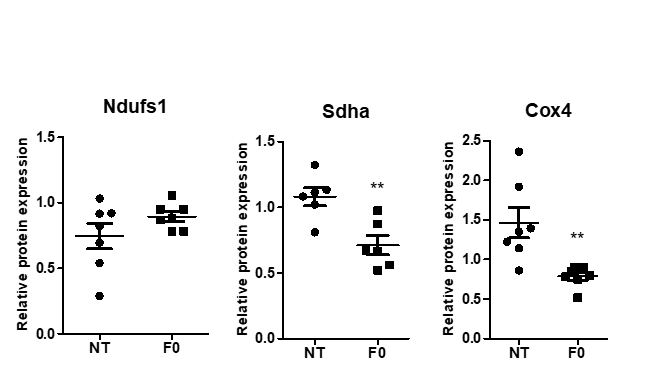


**Supplementary Figure S6:** Quantification of the levels of OXPHOS complex subunits from at least 3 independent experiments (N=3, n=6 or 7) accompanying Figure 3F.


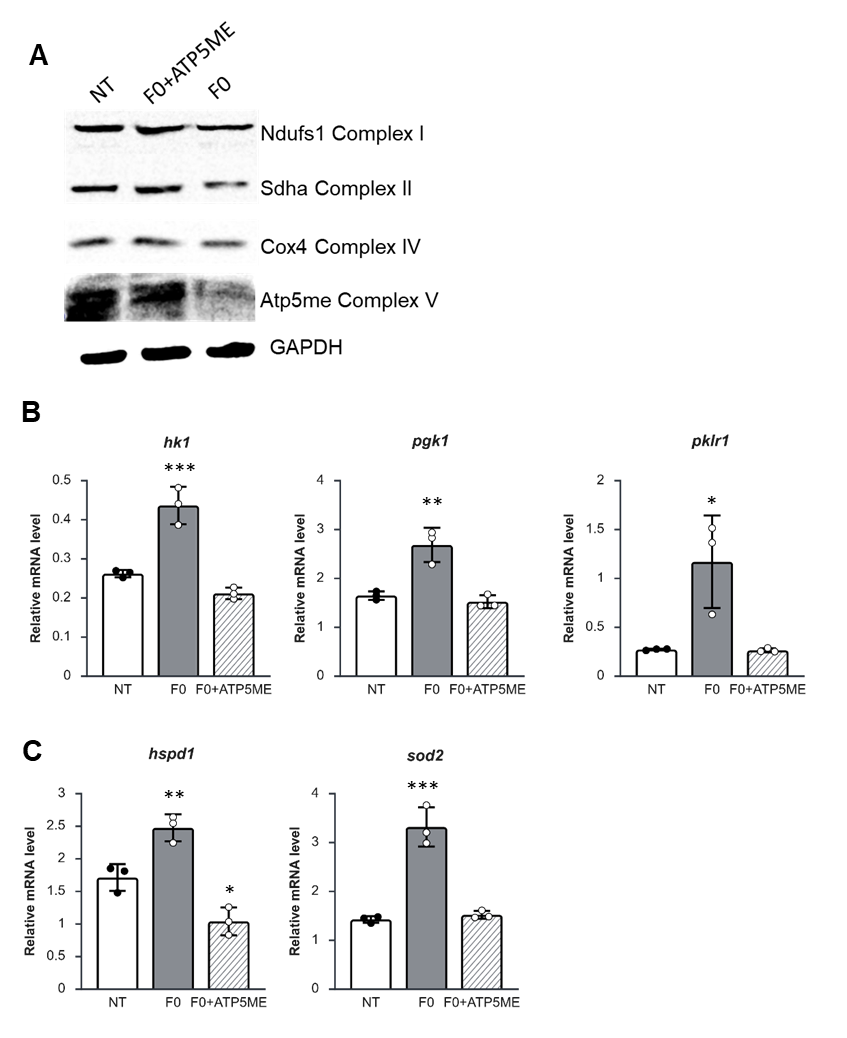


**Supplementary Figure S7: Rescue of phenotypes upon supplementation of human ATP5ME mRNA** **(A)** Measurement of protein levels of the subunits of the OXPHOS complexes in control, crispants and crispants injected with *ATP5ME* mRNA, by immunoblotting. **(B, C).** Relative mRNA levels of glycolysis pathways genes and oxidative stress markers in control, Atp5me crispants and crispants injected with *ATP5ME* mRNA (n=3/group).

**References**

1. Livak KJ, Schmittgen TD. Analysis of relative gene expression data using real-time quantitative PCR and the 2(-Delta Delta C(T)) Method. *Methods*. 2001;25(4):402-408.

2. Spinazzi M, Casarin A, Pertegato V, *et al*. Optimization of respiratory chain enzymatic assays in muscle for the diagnosis of mitochondrial disorders. *Mitochondrion*. 2011;11(6):893-904.

3. Wilmer MJ, van den Heuvel LP, Rodenburg RJ, *et al*. Mitochondrial complex V expression and activity in cystinotic fibroblasts. *Pediatr Res*. 2008;64(5):495-497.

4. Kuthethur R, Shukla V, Mallya S, Adiga D, Kabekkodu SP, Ramachandra L, Saxena PUP, Satyamoorthy K, Chakrabarty S. Expression analysis and function of mitochondrial genome-encoded microRNAs. J Cell Sci. 2022 Apr 15;135(8):jcs258937. doi: 10.1242/jcs.258937. Epub 2022 Apr 19. PMID: 35297485.

5. Timón-Gómez A, Pérez-Pérez R, Nyvltova E, Ugalde C, Fontanesi F, Barrientos A. Protocol for the Analysis of Yeast and Human Mitochondrial Respiratory Chain Complexes and Supercomplexes by Blue Native Electrophoresis. STAR Protoc. 2020 Sep 18;1(2):100089. doi: 10.1016/j.xpro.2020.100089. Epub 2020 Sep 3. PMID: 32995753; PMCID: PMC7521667.

6. Kimmel CB, Ballard WW, Kimmel SR, Ullmann B, Schilling TF. Stages of embryonic development of the zebrafish. Dev Dyn. 1995 Jul;203(3):253-310. doi: 10.1002/aja.1002030302. PMID: 8589427.
